# The role of microglial exosomes in brain injury

**DOI:** 10.3389/fncel.2022.1003809

**Published:** 2022-11-08

**Authors:** Yahong Chen, Jie Zhu, Jingjing Ji, Zhifeng Liu, Guangli Ren

**Affiliations:** ^1^Department of Pediatric, General Hospital of Southern Theater Command of PLA, Guangzhou, China; ^2^Graduate School, Southern Medical University, Guangzhou, China; ^3^Department of Critical Care Medicine, General Hospital of Southern Theater Command of PLA, Guangzhou, China

**Keywords:** microglia, exosomes, brain injury, regulation, proliferation, apoptosis

## Abstract

Microglia are involved in immune responses to central nervous system (CNS) injury. Meanwhile, exosomes derived from microglia are important mediators of information and material exchange in brain, which play an important role in neuroprotective or damaging effects. Microglial exosomes contain a variety of molecular cargos, including microRNAs, soluble proteins, and lipids, which have regulatory effects on other types of cells and microenvironment in brain. In this review, we summarized microglial exosome characteristics, release patterns, pro-proliferative and pro-apoptotic effects on neurons and other glial cells, immunomodulatory effects, and regulation of the extracellular microenvironment. Understanding the relationship between microglia exosomes and brain injury can provide new targets for clinical treatment.

## Background

Microglia are derived from primitive myeloid progenitor cells in the yolk sac and are important resident innate immune cells in the central nervous system (CNS), which participated in neuroinflammation and neurodegeneration spreading (Priller and Prinz, 2019). Rahimian et al. (2018, 2019a,b,2021) have concluded that activated microglia not only secreted TNF-α, IL-1β, ROS, etc. to promote neuroinflammation in cerebral ischemia but also secreted IL-4, IL-10, and TGF-β to inhibit neuroinflammation and repair tissue. Galectin-3(Gal-3) played a key role in the activation and proliferation of microglia, which regulated angiogenesis, neurogenesis, and inflammation after stroke by activating several different signaling pathways, including IL-4, IGF-1, and Ga^2+^. In summary, they play a bidirectional role in neuroinflammation, as well as antigen presentation and phagocytosis along with the morphological changes in microglia. Meanwhile, they communicated with diverse cellular populations with different ways in brain injury, including cytokines, exosomes, and endocytosis. It is involved in the occurrence and development of nerve injury and repair and plays a crucial role in the survival of neurons, the establishment of neural circuits, and the maintenance of the homeostasis of the brain microenvironment.

Exosomes, a type of extracellular vesicles, are lipid bilayer-wrapped spherical structures with a diameter of 30–150 nm (Li et al., 2021). A variety of cells can secrete exosomes, including platelets, lymphocytes, adipocytes, muscle cells, tumor cells, glial cells, neurons, and stem cells (Crenshaw et al., 2019; Davis, 2016). They can also be detected in biological fluids such as semen, saliva, plasma, urine, milk, cerebrospinal fluid (CSF), amniotic fluid, and tumor effusion (Camussi et al., 2011; Davis, 2016; Crenshaw et al., 2019). Exosomal cargoes vary a lot under different stimuli (Li et al., 2021). Correspondingly, microglial exosomes can be released into microenvironment and be uptaken by neighboring cells that participate in the regulation of other cell functions and extracellular microenvironment (Osier et al., 2018; Wortzel et al., 2019). They not only promote neuronal apoptosis or proliferation but also have the functions of immunomodulation. Importantly, exosomes can cross the blood–brain barrier (BBB) and be detected in the CSF and serum, thus leading to the possibility that may be useful for the diagnosis and treatment of CNS injury.

## Modulation of microglial exosomes release in brain injury

**FIGURE 1:**
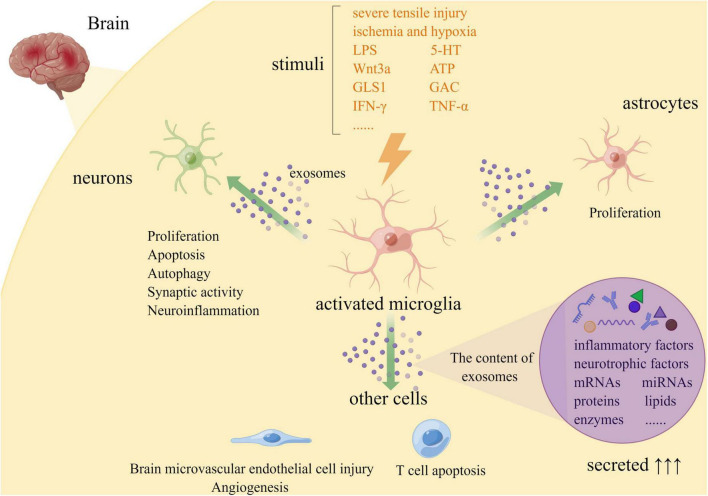
Release and role of microglial exosomes in brain injury (By Figdraw. ID:YAASUe13b4).

In general, exosomes need to be classified on the limiting membrane of multivesicular endosomes (MVEs) receptors according to their contents and then target MVEs to the plasma membrane, inward budding and fission generate intraluminal vesicles (ILVs) and release into the extracellular environment to be internalized by target cells (van Niel et al., 2018). The number of exosomes increased significantly and the contents of exosomes varied under external stimuli (Figure 1). Increasing evidence shows that exosomes derived from microglia increased significantly in response to stimuli, including severe tensile injury (Zhao et al., 2021), lipopolysaccharide (LPS) (Zhao et al., 2021), ischemia and hypoxia (Xie et al., 2020), 5-HT (Glebov et al., 2015), Wnt3a (Hooper et al., 2012), Glutaminase 1 (GLS1) (Gao et al., 2020), Glutaminase C (GAC) (Gao et al., 2019), ATP (Turola et al., 2012), IFN-γ (Hou et al., 2020), and TNF-α (Van den Broek et al., 2020). Among them, Glebov et al. (2015) suggested that 5-HT receptors, including 5-HT2a, 5-HT2b, and 5-HT4Rs may activate two different pathways that converge on phospholipase C (PLC), namely the G-PLC-IP3/DAG pathway. 5-HT4R can target adenylyl cyclase (AC) directly or interact with PLC through cAMP-mediated activation of epac1/2/Rap1 in microglia. Furthermore, neurons and other glial cells in the brain can also release exosomes to activate microglia, causing them to secrete corresponding exosomes (Hou et al., 2020; Borst et al., 2021). However, TNF played a key role in the regulation of microglial exosome release. LPS stimulated microglia activation, which produced a 30-fold increase in extracellular vesicles (EVs), but a 16-fold decrease in the number of EVs released after inhibition of TNF signaling (Yang et al., 2018). Meanwhile, previous studies found that cAMP and Ca^2+^-dependent signaling pathways are involved in the regulation of exosome secretion. Therefore, blocking the release of microglial exosomes or clearing harmful exosomes through PLC pathway, cAMP, and Ca^2+^-dependent signaling pathways may effectively delay the occurrence and development of brain injury, which is beneficial to the recovery of the disease.

## The role of microglial exosomes on cell proliferation and apoptosis in brain injury

Microglia secrete exosomes containing various miRNAs and interact with neurons or other glial cells to promote their proliferation, degeneration, or apoptosis (Figure 1).

### Involved in neuronal survival

Neurons, the effector cells in the brain, can receive, process, and transmit information to target cells in the CNS through axons and synapses. It plays an important role in the formation of a neural network (Liu et al., 2017). Therefore, inhibiting the apoptosis of neurons will help reduce brain injury and promote disease recovery. Studies have shown that microglia are activated after cerebral ischemia, and upregulation of GLS1 led to an increase in pro-inflammatory exosomes, causing neuroinflammatory damage to neurons (Turola et al., 2012). Current studies have confirmed that former M2 microglia-derived exosomal miR-124 can reduce neuronal apoptosis and relieve brain injury after stroke by downregulating ubiquitin-specific protease 14 (USP14) (Song et al., 2019) and regulation of Bcl-2/Bcl-xl pathway (Yu et al., 2017). It has been reported that ischemic neurons can also take up miRNA-137-containing exosomes through the Notch1 pathway to maintain their own survival (Zhang et al., 2021). Wei et al. (2021) demonstrated that microglia activated by intracerebral hemorrhage (ICH) inhibited the expression of activating transcription factor 4 (ATF4) by secreting miR-383-3p-containing exosomes, thereby promoting neuronal necroptosis. On the other hand, neuron-derived exosomes can also promote microglial polarization to mediate neuroinflammation. Furthermore, Ge et al. (2020) demonstrated that microglial exosomes with upregulated miR-124-3p contribute to alleviating neurodegeneration and promoting neurite growth in repetitive mild traumatic brain injury (rmTBI) mice by targeting Rela/ApoE signaling pathway in hippocampal neurons.

### Regulates the proliferation of astrocytes

Microglia can also affect neuronal function by regulating the proliferation of astrocytes. Ye et al. (2021) found that microglia can communicate with astrocytes through exosomes. Among them, the upregulation of microglial exosomal miRNA-145-5p can inhibit the proliferation of astrocytes in the early stage of injury by inhibiting the activity of smad3 and avoid excessive proliferation to form “glial scars,” which is conducive to the recovery of nerve function.

### Proliferation and apoptosis of other cells

As a novel way, microglia communicate with other cells via exosomes in the CNS. Xie et al. demonstrated that microglial exosomal miR-424-5p upregulation under hypoxia could induce brain microvascular endothelial cell (BMEC) injury by targeting FGF2-mediated STAT3 pathway (Xie et al., 2020). Besides, some studies have indicated that under high pressure, the exosomes of microglia can transmit denaturation signals *via* autocrine, leading to oxidative stress and cell death (Aires et al., 2020). We also found that microglia also interacted with oligodendrocytes in diseases such as Alzheimer’s disease, multiple sclerosis, amyotrophic lateral sclerosis, schizophrenia, and depression. However, the effects of microglial exosomes on oligodendrocyte were still unclear and need more in-depth study (Rahimian et al., 2022). In conclusion, microglial exosomes with different contents provide a new view of the treatment for brain injury.

## Immunomodulatory effects of microglial exosomes in brain injury

Existing studies show that microglia regulate immune response through phagocytosis and secretion of inflammatory mediators (Borst et al., 2021) or exosomes (Kalluri and LeBleu, 2020). Based on their roles in inflammation, microglia were divided into resting type, pro-inflammatory type (former M1 type), and anti-inflammatory type (former M2 type). They contacted target cells by secreting different types of inflammatory factors. However, whether these inflammatory factors were secreted directly by cells into the environment or capsuled in exosomes remains unclear. In ischemic stroke, microglia were activated to anti-inflammatory phenotype and subsequently transition to a pro-inflammatory phenotype at a later stage, achieving a balance of inflammatory responses (Jiang et al., 2020). We can distinguish former M1 and M2 subtypes by biomarkers. M1 markers include IL-1β, IL-6, IL-12 p70, TNF, IFNγ, CCL5, CCL20, CXCL1, CXCL10, GM-CSF, CD16, CD32, CD86, and iNOS. M2 markers include IL-1Ra, IL-4, IL-10, IL-4Rα, TGF-β, CCL22, CD206, CD163, Arg1, Ym1, FIZZ1, and G-CSF (Lan et al., 2017). However, with the in-depth study, more and more researches indicated that it is not sufficient to simply classify microglia into M1 and M2 phenotypes. One of the characteristic features of microglia is their transformation in response to CNS pathology. With the development of single-cell sequencing technology, the phenotype of microglia in brain injury was further revealed (Prinz et al., 2019). These studies have also furthered our understanding of the role of microglia in brain injury. Different phenotypes of microglia exosomes play different roles in brain injury due to their different contents, and we also summarized the roles of pro-inflammatory and anti-inflammatory phenotypes of microglia exosomes (Ransohoff, 2016).

### Phenotypic transformation of microglia

#### Pro-inflammatory phenotype

Lipopolysaccharide (LPS) induces polarization of microglia into pro-inflammatory subtype through activation of TLR2/TLR4/NF-kB/inflammasome signaling cascade and release of HMGB1 (Cunha et al., 2016). Under pathological conditions, microglia transformed into pro-inflammatory phenotype through downregulation of miR-124 to promote neuroinflammation (Yang et al., 2019). These microglia aggravate brain injury by releasing pro-inflammatory and neurotoxic factors including IL-1β, TNF-α, and IFN-γ (Zhao et al., 2020). Cunha et al. (2016) found that the expression profile of inflammation-miRs in exosomes was consistent with the expression profile of inflammation-miRs found in cells. That is to say, pro-inflammatory subtype microglia can directly transport soluble inflammatory signals and miRNAs to adjacent cells through exosomes.

#### Anti-inflammatory phenotype

Huang et al. (2018) demonstrated that miR-124-3p promoted microglial transforming to anti-inflammatory subtype by targeting PDE4B to inhibit the activity of the mTOR signaling pathway and suppress neuronal inflammation in scratched neurons. Furthermore, Yu et al. (2017) found that miR-124 can also attenuate inflammatory responses via modulating transcription factor C/EBP-α. At the same time, Long et al. (2020) discovered activated astrocytes secrete exosomes rich in miR-873a-5p, which promoted transformation of anti-inflammatory microglia by inhibiting phosphorylation of ERK and NF-κB p65, thereby alleviating neurological deficits after traumatic brain injury (TBI).

### Effects of microglial exosomes on immune cells

Disease-associated microglia play a key role in neuroinflammation, while they can also influence multiple immune cell functions through exosomes (Prinz et al., 2019). Some studies have found that exosomal IL-10 and FASL signaling can induce T cell apoptosis, which is beneficial to the amplification of regulatory T cells and myeloid suppressor cells. Exosomes can increase the activity of natural killer (NK) cells and the survival rate of T cells, on the one hand, and inhibit the activity of NK cells, T cell proliferation, and dendritic cell (DC) differentiation, on the other hand, thus exerting a bidirectional immunomodulatory effect (El Andaloussi et al., 2013). It has been reported that microglia can also secrete NAMPT through exosomes during neuroinflammation of ischemic injury and participate in the inflammatory response (Yang et al., 2019). Several studies have shown that in brain injury such as TBI and transient middle cerebral artery occlusion (tMCAO), the treatment of microglial exosomes can reduce neuroinflammation and immune responses (Huang et al., 2018; Song et al., 2019). Taken together, microglial exosomes are involved in the immune regulation of brain injury.

## Regulation of the extracellular microenvironment

### Affects neurotransmitter transmission

Exosomes secreted by microglia can affect neurotransmitter transmission. It has been reported that microglial exosomes are involved in the homeostatic regulation of neurotransmission by enhancing spontaneous and evoked excitatory transmission through interacting with neuronal plasma membranes and neuronal sphingolipid metabolism (Turola et al., 2012). Some studies have found that exosomes secreted by microglia can inhibit presynaptic transmission by binding to receptors, which is due to the endocannabinoid N-arachidonoylethanolamine (AEA) carried on its surface. Exosomes carrying AEA can stimulate the type 1 cannabinoid (CB1) receptor expressed by GABAergic neurons, thereby inhibiting presynaptic transmission (Gabrielli et al., 2015).

### Maintain synaptic activity

Microglia can prune synapses and maintain synaptic homeostasis through phagocytosis of synapses, which play an important role in establishing neural circuits and maintaining the function of nerve cells. Exosomes derived from microglia can also transport enzymes and other substances, promote metabolism such as anaerobic glycolysis and lactate production, and supplement energy for synaptic activities (Pistono et al., 2020). In addition, studies have shown that injured microglia may affect synaptic activity by downregulating exosomal miR-5121 and transmitting signals to stretch injured neurons after TBI, inhibiting the growth of some neurites and synaptic recovery (Zhao et al., 2021).

### Angiogenesis

Tian et al. (2019) found that IL-4-polarized BV2 cells may promote angiogenesis by secreting exosomes containing miR-26a. Subsequently, it was also confirmed that former M2 microglia can secrete exosomes to promote neovascularization, and HIF pathway may be involved. The delivery of oxygen, nutrients, and even immune cells to the injured site through neovascularization will facilitate the recovery of the disease. For example, the ischemic area promotes the recovery of ischemic stroke due to the entry of more Th2 or former M2 cells.

### Maintain cellular homeostasis

Microglia exosomes can also maintain cellular homeostasis and repair functions. They can activate autophagy through the formation of LC3B-positive autophagosomes in microglia (Van den Broek et al., 2020). For example, microglia-derived exosomes activated by exosomes from SH-SY5Y cells overexpressing α-synuclein can target PTEN through miR-19a-3p, thereby activation of the PI3K/Akt/mTOR signaling pathway ultimately inhibits neuronal autophagy. Other studies have shown that inhibition of autophagy can induce former M1 microglia polarization and the release of TNF-α, iNOS, and Cyclooxygenase 2 (Cox2), which can aggravate the injury of brain (Xia et al., 2016). Thus, microglial exosomes maintain cellular homeostasis by activating autophagy.

## Conclusion and prospect

We have summarized the most recent evidence regarding microglia-derived exosomes playing an important role in brain injury due to changes in the type of cargoes. Microglial exosomes, as important information transfer mediators, can communicate with a variety of cells in the brain, including neurons. Meanwhile, they can affect the microenvironment, then affecting neurological function and blood–brain barrier integrity. Some studies already suggest that microglia can influence CNS function through miRNAs, neurotransmitters, and proteins in exosomes, and many types of bioactive substances in exosomes can be used as disease biomarkers of brain injury.

The authors of this review consider that analysis of microglia exosome release, delivery, and contents will provide a more comprehensive and accurate assessment of brain injury development. The information about blocking, promoting, and modifying exosomal contents will provide a safer and robust line of treatment.

## Author contributions

YC, JZ, and JJ: drafting and refining the manuscript. ZL and GR: critical reading of the manuscript. All authors read and approved the manuscript.
